# Supplementary material for: The Effect of Non-Pharmacological and Pharmacological Interventions on Measures Associated with Sarcopenia in End-Stage Kidney Disease: A Systematic Review and Meta-Analysis
Source: Nutrients. 2022 Apr 27;14(9):1817. doi: 10.3390/nu14091817 (PMC9101978; doi:10.3390/nu14091817)
Supplement: Supplementary file 1 [file nutrients-14-01817-s001.zip › nutrients-1660678-supplementary.pdf]

Table S1: Full search strategy for the MEDLINE and CINAHL databases. Dual-energy x-ray absorptiometry (DEXA), end-stage kidney disease (ESKD), end-stage renal disease (ESRD), magnetic resonance imaging (MRI), MeSH terms (MH), short physical performance battery (SPPB).

	Search term	Field
1	Renal insufficiency	MH (explode)
2	Renal insufficiency	TX
3	Renal insufficiency, chronic	MH (explode)
4	Renal insufficiency, chronic	TX
5	Renal Dialysis	MH (explode)
6	Renal dialysis	TX
7	Kidney failure, chronic	MH
8	Kidney failure, chronic	TX
9	Renal Replacement therapy	MH
10	Renal Replacement therapy	TX
11	Haemodialysis	TX
12	Hemodialysis	TX
13	Dialysis	TX
14	ESRD	TX
15	ESKD	TX
16	kidney disease adj2 (end-stage OR end stage OR chronic)	TX
17	renal disease adj2 (end-stage OR end stage OR chronic)	TX
18	kidney failure adj2 (end-stage OR end stage OR chronic)	TX
19	Renal replacement therapy	TX
20	renal failure adj2 (end-stage OR end stage OR chronic)	TX
21	Transplants	MH (explode)
22	Transplants	TX
23	Kidney diseases	MH (explode)
24	Kidney diseases	TX
25	Peritoneal dialysis	MH (explode)
26	Peritoneal dialysis	TX
27	Peritoneal dialysis, continuous ambulatory	MH (explode)
28	Peritoneal dialysis, continuous ambulatory	TX
29	Kidney transplantation	MH (explode)
30	Kidney transplantation	TX
31	Conservative treatment	MH (explode)
32	Conservative treatment	TX
33	1 OR 2 OR 3 OR 4 OR 5 OR 6 OR 7 OR 8 OR 9 OR 10 OR 11 OR 12 OR 13 OR 14 OR 15 OR 16 OR 17 OR 18 OR 19 OR 20 OR 21 OR 22 OR 23 OR 24 OR 25 OR 26 OR 27 OR 28 OR 29 OR 30 OR 31 OR 32 OR 32	
34	Life Style	MH (explode)
35	Life Style	TX
36	Lifestyle	TX
37	Lifestyle intervention	TX
38	Exercise	MH (explode)
39	Exercise	TX
40	Healthy lifestyle	MH (explode)
41	Healthy lifestyle	TX
42	Exercise therapy	MH (explode)
43	Exercise therapy	TX
44	Exercise training	TX
45	Exercise program*	TX
46	Aerobic exercise	TX
47	Aerobic training	TX
48	Resistance training	MH (explode)
49	Resistance training	TX
50	Physical Activity	TX
51	Diet	MH (explode)
52	Diet	TX
53	Nutrition	TX
54	Nutrition therapy	MH (explode)
55	Nutrition therapy	TX
56	Non adj2 pharmacological	TX
57	Pharmaceutical preparations	MH (explode)
58	Pharmaceutical preparations	TX
59	Pharmaceutical Aids	MH (explode)
60	Pharmaceutical Aids	TX
61	Pharmacological intervention	TX
62	Pharmaceutical	TX
63	Drug	TX
64	Vitamin adj2 d	TX
65	Growth adj2 hormone	TX
66	Dehydroepiandrosterone	TX
67	Growth adj2 factor	TX
68	Testosterone	TX

**69**	Angiotensin-Converting Enzyme Inhibitors	MH (explode)
**70**	Angiotensin-Converting Enzyme Inhibitors	TX
**71**	ACE inhibitor	TX
**72**	34 OR 35 OR 36 OR 37 OR 38 OR 39 OR 40 OR 41 OR 42 OR 43 OR 44 OR 45 OR 46 OR 47 OR 48 OR 49 OR 50 OR 51 OR 52 OR 53 OR 54 OR 55 OR 56 OR 57 OR 58 OR 59 OR 60 OR 61 OR 62 OR 63 OR 64 OR 65 OR 66 OR 67 O 68 OR 69 OR 70 OR 71	
**73**	Sarcopenia	MH (explode)
**74**	Sarcopenia	TX
**75**	Cachexia	MH (explode)
**76**	Cachexia	TX
**77**	Muscle adj2 wasting	TX
**78**	Protein-energy malnutrition	MH (explode)
**79**	Protein-energy malnutrition	TX
**80**	Wasting adj2 (protein energy OR protein-energy)	TX
**81**	Frailty	MH (explode)
**82**	Frailty	TX
**83**	Muscle weakness	MH (explode)
**84**	Muscle weakness	TX
**85**	Muscle atrophy	MH (explode)
**86**	Muscle atrophy	TX
**87**	Muscle strength	MH (explode)
**88**	Muscle strength	TX
**89**	Body composition	TX
**90**	Fat free mass	TX
**91**	Muscle, skeletal	MH (explode)
**92**	Muscle, skeletal	TX
**93**	Lean body mass	TX
**94**	Muscle mass	TX
**95**	Grip strength	TX
**96**	Sit adj2 stand	TX
**97**	MRI	TW
**98**	Magnetic resonance imaging	TW
**99**	DEXA	TW
**100**	Dual-energy x-ray absorptiometry	TX
**101**	Bioelectrical adj2 impedance	TX
**102**	CT adj2 imaging	TW
**103**	Physical functional performance	MH (explode)
**104**	Physical functional performance	TX
**105**	Short physical performance battery	TX
**106**	SPPB	TX
**107**	Timed up and go	TX
**108**	Walk adj2 speed	TX
**109**	Gait adj2 speed	TX
**110**	Quality adj2 life	TW
**111**	Physical adj2 activity	TW
**112**	Fall*	TW
**113**	Hospital adj2 admissions	TW
**114**	73 OR 74 OR 75 OR 76 OR 77 OR 78 OR 79 OR 80 OR 81 OR 82 OR 83 OR 84 OR 85 OR 86 OR 87 OR 88 OR 89 OR 90 OR 91 OR 92 OR 93 OR 94 OR 95 OR 96 Or 97 OR 98 OR 99 OR 100 OR 101 OR 102 OR 103 OR 104 OR 105 OR 106 OR 107 OR 108 OR 109 OR 110 OR 111 OR 112 OR 113	
**115**	Randomized controlled trial as topic	MH (explode)
**116**	Controlled clinical trial	MH (explode)
**117**	Randomized OR Randomised	TX
**118**	Placebo	TX
**119**	Drug therapy	TX
**120**	Random*	TX
**121**	Trial	TX
**122**	Groups	TX
**123**	115 OR OR 116 OR 117 OR 118 OR 119 OR 120 OR 121 OR 122	TX
**124**	Exp animals/ not humans	TX
**125**	123 NOT 124	TX
**126**	33 AND 72 AND 114 AND 125	

Table S2: Full search strategy for EMBASE database. All fields (AF), End-stage kidney disease (ESKD), end-stage renal disease (ESRD).

	Search term	Field
**1**	Renal insufficiency	AF
**2**	Renal insufficiency, chronic	AF
**3**	Renal Dialysis	AF
**4**	Kidney failure, chronic	AF
**5**	Renal Replacement therapy	AF
**6**	Haemodialysis	AF
**7**	Hemodialysis	AF
**8**	Dialysis	AF
**9**	ESRD	AF
**10**	ESKD	AF
**11**	kidney disease adj2 (end-stage OR end stage OR chronic)	AF
**12**	renal disease adj2 (end-stage OR end stage OR chronic)	AF
**13**	kidney failure adj2 (end-stage OR end stage OR chronic)	AF
**14**	Renal replacement therapy	AF
**15**	renal failure adj2 (end-stage OR end stage OR chronic)	AF
**16**	Transplant*	AF
**17**	Kidney diseases	AF
**18**	Peritoneal dialysis	AF
**19**	Peritoneal dialysis, continuous ambulatory	AF
**20**	Kidney transplantation	AF
**21**	Conservative treatment	
**22**	1 OR 2 OR 3 OR 4 OR 5 OR 6 OR 7 OR 8 OR 9 OR 10 OR 11 OR 12 OR 13 OR 14 OR 15 OR 16 OR 17 OR 18 OR 19 OR 20 OR 21	
**23**	Lifestyle	AF
**24**	Lifestyle intervention	AF
**25**	Exercise	AF
**26**	Healthy Lifestyle	AF
**27**	Exercise therapy	AF
**28**	Exercise training	AF
**29**	Exercise program*	AF
**30**	Aerobic exercise	AF
**31**	Aerobic training	AF
**32**	Resistance training	AF
**33**	Physical Activity	AF
**34**	Diet	AF
**35**	Nutrition	AF
**36**	Nutrition therapy	AF
**37**	Non adj2 pharmacological	AF
**38**	Pharmaceutical preparations	AF
**39**	Pharmaceutical Aids	AF
**40**	Pharmacological intervention	AF
**41**	Pharmaceutical	AF
**42**	Drug	AF
**43**	Vitamin adj2 d	AF
**44**	Growth adj2 hormone	AF
**45**	Dehydroepiandrosterone	AF
**46**	Growth adj2 factor	AF
**47**	Testosterone	AF
**48**	Angiotensin-Converting Enzyme Inhibitors	AF
**49**	ACE inhibitor	AF
**50**	23 OR 24 OR 25 OR 26 OR 27 OR 28 OR 29 OR 30 OR 31 OR 32 OR 33 OR 34 OR 35 OR 36 OR 37 OR 38 OR 39 OR 40 OR 41 OR 42 OR 43 OR 44 OR 45 OR 46 OR 47 OR 48	
**51**	Sarcopenia	AF
**52**	Cachexia	AF
**53**	Muscle adj2 wasting	AF
**54**	Protein-energy malnutrition	AF
**55**	Wasting adj2 (protein energy or protein-energy)	AF
**56**	Frailty	AF
**57**	Muscle weakness	AF
**58**	Muscle atrophy	AF
**59**	Muscle strength	AF
**60**	Body composition	AF
**61**	Fat free mass	AF
**62**	Skeletal muscle	AF
**63**	Lean body mass	AF
**64**	Muscle mass	AF
**65**	Grip strength	AF

**66**	51 OR 52 OR 53 OR 54 OR 55 OR 56 OR 57 OR 58 OR 59 OR 60 OR 61 OR 62 Or 63 OR 64 OR 65	
**67**	Clinical Trial	AF
**68**	Randomized controlled trial	AF
**69**	Randomised controlled trial	AF
**70**	Random allocation	AF
**71**	Randomised	AF
**72**	Randomized	AF
**73**	Randomly	AF
**74**	Allocated adj2 random	AF
**75**	Random adj3 crossover	AF
**76**	Randomised or randomized	AF
**77**	Trial	AF
**78**	Group	AF
**79**	66 OR 67 OR 68 OR 69 OR 70 OR 71 OR 72 OR 73 OR 74 OR 75 OR 76 OR 77	
**80**	22 AND 50 AND 66 AND 79	

	Whey Protein			Control				Mean Difference
**Study or Subgroup**	**Mean**	**SD**	**Total**	**Mean**	**SD**	**Total**	**Weight**	**IV, Random, 95% CI**
Jeong et al., 2019	58.6	15.9	38	60.4	12.4	34	16.9%	-1.80 [-8.35, 4.75]
Tomayko et al., 2015	57.6	3.8	11	59.1	3.8	15	83.1%	-1.50 [-4.46, 1.46]
**Total (95% CI)**			**49**			**49**	**100.0%**	**-1.55 [-4.25, 1.14]**

Heterogeneity: Tau² = 0.00; Chi² = 0.01, df = 1 (P = 0.93); I² = 0%
Test for overall effect: Z = 1.13 (P = 0.26)

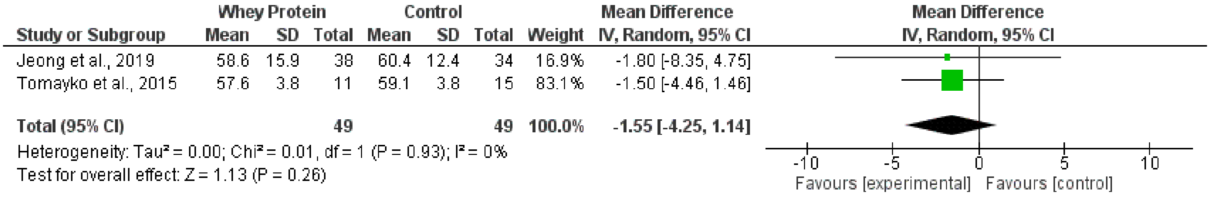

Figure S1. Effect of whey protein supplementation on whole lean body mass (kg) measured by DEXA in individuals receiving haemodialysis. Data are expressed as mean difference and 95% CI. Data for whey protein and control groups from both trials [22,66].

	Whey Protein			Control				Mean Difference
**Study or Subgroup**	**Mean**	**SD**	**Total**	**Mean**	**SD**	**Total**	**Weight**	**IV, Random, 95% CI**
Jeong et al., 2019	8	3.6	38	8.2	5.1	34	14.7%	-0.20 [-2.26, 1.86]
Tomayko et al., 2015	7.6	1.1	11	8.2	1.1	15	85.3%	-0.60 [-1.46, 0.26]
**Total (95% CI)**			**49**			**49**	**100.0%**	**-0.54 [-1.33, 0.25]**

Heterogeneity: Tau² = 0.00; Chi² = 0.12, df = 1 (P = 0.73); I² = 0%
Test for overall effect: Z = 1.34 (P = 0.18)

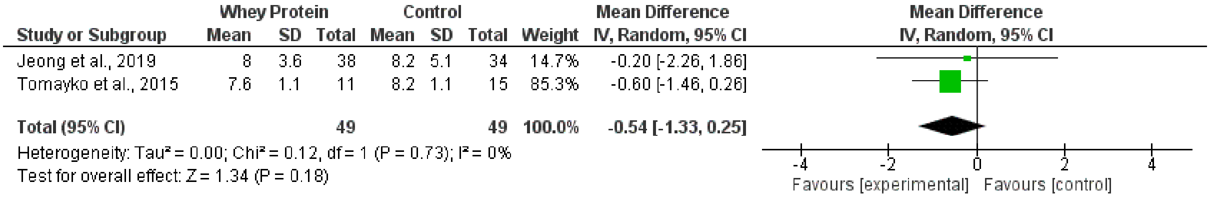

Figure S2. Effect of whey protein supplementation on timed-up-and go in individuals receiving haemodialysis. Data are expressed as mean difference and 95% CI. Data for whey protein and control groups from both trials[22,66].
